# Hearing to the Unseen: AudioMoth and BirdNET as a Cheap and Easy Method for Monitoring Cryptic Bird Species

**DOI:** 10.3390/s23167176

**Published:** 2023-08-15

**Authors:** Gerard Bota, Robert Manzano-Rubio, Lidia Catalán, Julia Gómez-Catasús, Cristian Pérez-Granados

**Affiliations:** 1 Conservation Biology Group, Landscape Dynamics and Biodiversity Programme, Forest Science and Technology Center of Catalonia (CTFC), 25280 Solsona, Spain; gerard.bota@ctfc.cat (G.B.); robert.manzano@ctfc.cat (R.M.-R.); 2 Independent Researcher, 44002 Teruel, Spain; lidiacrispi@gmail.com; 3 Terrestrial Ecology Group (TEG-UAM), Department of Ecology, Autonomous University of Madrid, 28049 Madrid, Spain; julia.gomez@uam.es; 4 Research Centre in Biodiversity and Global Change (CIBC-UAM), Autonomous University of Madrid, 28049 Madrid, Spain; 5 Ecology Department, Alicante University, 03080 Alicante, Spain

**Keywords:** acoustic sensor, audio recognition, automated recognition software, autonomous recording unit, machine learning, Paridae, *Periparus ater*, passive acoustic monitoring, wildlife monitoring

## Abstract

The efficient analyses of sound recordings obtained through passive acoustic monitoring (PAM) might be challenging owing to the vast amount of data collected using such technique. The development of species-specific acoustic recognizers (e.g., through deep learning) may alleviate the time required for sound recordings but are often difficult to create. Here, we evaluate the effectiveness of BirdNET, a new machine learning tool freely available for automated recognition and acoustic data processing, for correctly identifying and detecting two cryptic forest bird species. BirdNET precision was high for both the Coal Tit (*Peripatus ater*) and the Short-toed Treecreeper (*Certhia brachydactyla*), with mean values of 92.6% and 87.8%, respectively. Using the default values, BirdNET successfully detected the Coal Tit and the Short-toed Treecreeper in 90.5% and 98.4% of the annotated recordings, respectively. We also tested the impact of variable confidence scores on BirdNET performance and estimated the optimal confidence score for each species. Vocal activity patterns of both species, obtained using PAM and BirdNET, reached their peak during the first two hours after sunrise. We hope that our study may encourage researchers and managers to utilize this user-friendly and ready-to-use software, thus contributing to advancements in acoustic sensing and environmental monitoring.

## 1. Introduction

Nowadays, there exists an increasing demand for automated, efficient, and scalable ecological monitoring methodologies that possess the capability to address the ongoing decline in biodiversity [1]. Conventional field surveys, which rely on human presence in the field, may suffer from limitations and biases stemming from human expertise, while also being time-consuming and costly [1,2]. Fortunately, advancements in sensing technologies and computational capabilities now enable the execution of automated ecological surveys on a large scale, both spatially and temporally. Innovative biomonitoring techniques (see [3]) decrease the need for human presence in the field while reducing potential biases associated with human-based surveys. Although new technologies provide important improvements over traditional monitoring methods, their application is not exempt from considerations and limitations. For example, the technology considered should be selected taking into account the goals of the biodiversity monitoring scheme, the indicators to be measured, the accuracy for target taxa, as well as the available capacity of and budget for equipment [4,5].

Many animal species, ranging from small insects to whales, emit acoustic signals, so acoustic communication is widespread in the animal world, and very often species communicate using a sequence of distinct acoustic elements [6]. One of the emerging noninvasive and automated techniques for ecological monitoring is passive acoustic monitoring (PAM). This method involves the utilization of Autonomous Recording Units (ARUs) equipped with acoustic sensors (referred to as microphones hereafter) that are deployed in the field to obtain recorded acoustic data using a specified recording schedule. ARUs are sound recorders that can be programmed with specific time schedules to be unattended while operating in the field, with great battery autonomy and storage capacity and built to operate in outdoor conditions [7]. The subsequent analysis of these recorded sounds enables the detection and monitoring of individuals or ecosystems without disrupting their natural behavior [2].

PAM is a trending technique whose use to monitor species and ecosystems is increasingly gaining more and more attention (see reviews in [2,8]). This rise in popularity can be partially attributed to several factors. Firstly, the availability of affordable and efficient ARUs, such as AudioMoth [9,10], has facilitated the widespread adoption of PAM. Secondly, recent innovations in acoustic data processing techniques [11,12] have enhanced the analysis and interpretation of acoustic data obtained through PAM. Lastly, the development of low-cost yet high-quality microphones (e.g., [13]) has further contributed to the advancement of PAM. Passive acoustic surveys generate a significant amount of data, posing challenges for visual or acoustical verification of the recordings (but, see [14]). To address this issue, a wide range of audio signal recognition tools have been developed over the past decade to assist with audio processing and enable fast and accurate interpretation of the extensive acoustic data obtained from passive acoustic surveys. These tools encompass a spectrum of approaches, ranging from basic detectors that employ template matching methods (e.g., [15]) to advanced techniques, like deep learning and convolutional neural networks, which represent the current state-of-the-art in the field [12].

Birds are the most commonly monitored group of animals using PAM [2] and, consequently, the majority of advances in audio signal recognition have been focused on birds (see [11,16,17]). State-of-the-art techniques have demonstrated their ability to develop highly accurate bird recognition models. However, these sophisticated methods may pose challenges for implementation by managers, scientists, and the general public due to the significant level of informatics experience required [17]. Fortunately, a recently updated machine learning tool called BirdNET provides a free and user-friendly solution for automated audio recognition [11]. This ready-to-use tool has been widely adopted by the general public (over 1.1 million participants used the BirdNET APP during 2020, [18]) and by scientists (see review of applications in [19]), enabling an easy access and use of machine learning for automated wildlife recognition. BirdNET employs a deep neural network for automated detection and classification of wildlife vocalizations [11]. The tool divides the original sound recordings into 3-s segments and provides identification for over 6000 species of wildlife for each segment [11,18]. BirdNET can identify multiple species within the same segment, and each detection is accompanied by a quantitative confidence score, automatically provided by BirdNET, ranging from 0 to 1. This score reflects the probability of accurately identifying the species, with a score of 1 indicating a perfect match. The confidence score can be adjusted by the user as a threshold value, enabling the filtering of BirdNET output at a desired confidence level. Selecting a higher confidence score increases the percentage of correctly classified detections but may result in a lower number of detections overall. However, our current knowledge on how confidence score impacts BirdNET species detection accuracy is very limited (reviewed in [19]). Additionally, BirdNET allows the users to adjust the overlap of prediction segments, modify the sensitivity parameter, and to apply filters to classify sounds based on recording location, time period, or target species [11,19].

BirdNET, as a user-friendly tool, can be easily accessed through various friendly interfaces. It can be used in a smartphone (BirdNET App, [18]), enabling users to directly record bird sounds in the field. Alternatively, a web-based platform called BirdNET-API allows users to upload their recordings for analysis [20]. BirdNET functionality is also integrated into Raven Pro, an audio software developed by the Cornell Lab of Ornithology, and can be run on Windows or Python through the BirdNET-Analyzer, which is openly accessible on GitHub (https://github.com/kahst/BirdNET-Analyzer, accessed on 11 August 2023). While BirdNET was initially designed for bird species recognition, the most recent updates have expanded its capabilities to include a limited number of other species, such as frogs and primates [21,22]. However, the extent of BirdNET’s ability in correctly identifying bird species vocalizations from sound recordings collected using omnidirectional microphones, the ones typically used in PAM, remains largely confined to a few case studies (as reviewed by [19]).

In this study, our objective is to conduct a comprehensive evaluation of the usefulness of using low-cost recorders (AudioMoth) and BirdNET for monitoring two cryptic forest bird species. For each species, we have set out the following aims: (1) Assessing the precision of BirdNET in correctly identifying bird vocalizations; (2) Determining the optimal confidence score threshold of each species, which might be useful to establish a reliable criterion for accepting BirdNET detections with a high level of confidence; (3) Estimating the percentage of presences automatically identified by BirdNET compared to human visual inspection of sonograms with the default values and using the optimal confidence score threshold; (4) Apply the method on a large field acoustic dataset aiming to describe the diel vocal behavior of the studied species, which we recorded during the daily period over the course of one month in two distinct habitat types. While our assessment is restricted to two bird species, we hope that our approach may be a valuable guidance to improve the overall quality of passive acoustic surveys using widely spread low-cost ARUs and free user-friendly automated audio processing software.

## 2. Materials and Methods

### 2.1. Study Species

The Coal Tit (*Periparus ater*) and the Short-toed Treecreeper (*Certhia brachydactyla*) were selected as the forest study bird species owing to their cryptic behavior and challenges for monitoring using visual cues. The Coal Tit is a small passerine that is resident in Europe, North Africa, and parts of Asia [23]. The distribution of the Short-toed Treecreeper is limited to Europe and North Africa [24]. The challenges associated with monitoring the Coal Tit are primarily related to its canopy habits, as they often inhabit the upper and outer parts of the forest canopy [25], making them potentially difficult to observe depending on forest structure. The Short-toed Treecreeper is a small passerine specialized in feeding on insects found in bark crevices. While its preference for feeding in trunks may make them more detectable, the genus *Certhia*, to which the Short-toed Treecreeper belongs, is characterized for its effective camouflage against tree trunks, making it challenging for humans to visually detect them in trunks [26]. Fortunately, both species exhibit high vocal activity. Male Coal Tits produce songs primarily during the breeding season, characterized by a common pattern of two or three ascending or descending elements that are repeated several times [23,27]. The song of the Short-toed Treecreeper is relatively simple but distinctive. It consists of a low-pitched vocalization that is repeated several times [28]. Given the high vocal activity of both species and the challenges associated with their visual detection, passive acoustic monitoring might be a reliable tool for monitoring their presence and providing valuable insights into their behavior.

### 2.2. Study Area

The study was carried out in two forest areas located in the Comunidad Foral de Navarra (Northern Spain). Both areas were located at about 600–800 m a.s.l. and separated around 30 km. The first forest was predominantly dominated by Sessile Oak (*Quercus petraea*), which was located in the municipality of Etxarri (42°58′10.96″ N, 1°53′6.33″ W). This forest is recognized as one of the well-preserved oak forests in Navarra. The second forested area, situated in the municipality of Aitzarotz (43°1′27.83″ N, 1°45′55.72″ W), was dominated by European Beech (*Fagus sylvatica*). The Navarra region experiences a humid and temperate climate, with average annual temperatures ranging between 8.5 and 14.5 °C and a typical annual rainfall regime between 1100 and 2500 mm.

### 2.3. Acoustic Monitoring Protocol

We deployed one AudioMoth recorder [9] in each monitored forest, which operated from 24 February to 23 March 2022. This period corresponds to the pre-breeding period for both species in temperate forests (first laying date around mid April, [29,30]). The recorders were securely housed in AudioMoth IPX7 cases (Open Acoustic Devices) and mounted on trees at a height of approximately 1.5 m above the ground. The recorders were configured to record audio at a sampling rate of 48 kHz, gain Med–High, and 16 bits per sample. The recording schedule spanned from 8 a.m. to 7 p.m., with a single 15-min recording captured at the beginning of each hour. This schedule was designed to encompass most of the daily hours, considering local sunrise occurring at 7:30 a.m. and sunset at 6:30 p.m. (sunrise and sunset estimates for 10 March in the study area). Following this protocol, we obtained a total of 11 15-min recordings per day and study location. This gave a total of 336 15-min recordings per site (87 h of recording per site) during the whole study period.

### 2.4. Recording Analyses

Acoustic recordings were analyzed using BirdNET (version 2.2.0, [11]). BirdNET was run using the default parameter values, including a sensitivity parameter of 1.0, a confidence score threshold of 0.1, and no overlap of prediction segments (0). We configured BirdNET to report detections exclusively for the Coal Tit and the Short-toed Treecreeper, therefore, avoiding the detection of nontarget species [31].

### 2.5. Recognizer Performance

We estimated the precision of BirdNET, a commonly used acoustic metric for assessing recognizer performance [32]. BirdNET precision was assessed without applying any filtering on the confidence score threshold. Precision was estimated as the proportion of BirdNET detections correctly classified by the total verified BirdNET detections [32]. To estimate precision, we randomly selected 309 BirdNET detections from the Coal Tit BirdNET output (28.5% of total detections) and 311 detections within the BirdNET output reported for the Short-toed Treecreeper (10.2% of total detections). We included a larger number of detections with lower confidence scores because there is a higher probability of mislabeling detections with lower confidence scores but also because there were a larger number of detections with low confidence scores in BirdNET’s output. For each selected detection, the observer listened to and inspected the spectrogram in Raven Pro 1.6 [33] at the timestamp of the 3-s segment and reported whether the target species was present or absent. This process allowed us to determine the proportion of BirdNET detections that were correctly classified among the total verified BirdNET detections [32].

We also used the validation dataset described in the paragraph above to identify the confidence score threshold with a 95% probability of correct identification for each species. Following the approach outlined in [22], we back-transformed BirdNET’s confidence scores into its original logit scale using the following equation:

Logit score = ln(1/(1 – confidence score))

Next, for each species, we fitted a logistic regression to establish a relationship between the correct or incorrect classification of the validated detections as a response variable and the BirdNET logit-scale prediction score as an independent variable. The logistic regressions provide an equation that enables us to convert BirdNET scores into the probability of a given prediction being correct. For each species, the equations considering a probability of correct identification of 95% were as follows:

logit(P) = ln(intercept) + 0.95 × ln(logit-score)

The identified optimal score was used as a confidence score threshold to consider only BirdNET detections with a high probability of correct identification when describing the diel pattern of vocal activity. The diel pattern of vocal activity was described showing the percentage of BirdNET detections made per recording hour and location for each of the monitored species.

Finally, we also assessed the detection and identification performance of BirdNET for each bird species, by establishing a validation dataset consisting of referenced recordings. This involved a manual review of 100 recordings, with 50 recordings from each location among those recorded between 8 a.m. and 9 a.m. For each recording, a human observer assessed whether the Coal Tit and/or the Short-toed Treecreeper were detected by visually inspecting spectrograms in Raven Pro 1.6 [33]. Recordings were reviewed blindly without knowledge of the site location, date, or time of recording. To assess the effectiveness of BirdNET in detecting the Coal Tit and the Short-toed Treecreeper, we determined the percentage of presences detected by BirdNET compared to the total number of recordings with known presence in the validation dataset. For this evaluation, we examined every audio recording that BirdNET annotated as containing one or both species. An expert observer verified, by listening to or inspecting the spectrogram, whether the species was truly present or absent at the timestamp of the 3-s segment annotated by BirdNET. If the species was absent at the selected timestamp, additional detections were verified until the presence of the species was confirmed or until the last detection was checked. Those recordings with no BirdNET annotations were annotated as absences according to BirdNET output. We estimated the percentage of occurrences detected by BirdNET in comparison to a human verification in two scenarios: (1) Without applying any filtering to BirdNET’s output (i.e., default values, all detections with confidence scores above 0.1 were included); (2) By filtering BirdNET’s output by the optimal confidence score threshold for each species (see Section 3).

## 3. Results

### 3.1. Recognizer Performance

**Figure 1:**
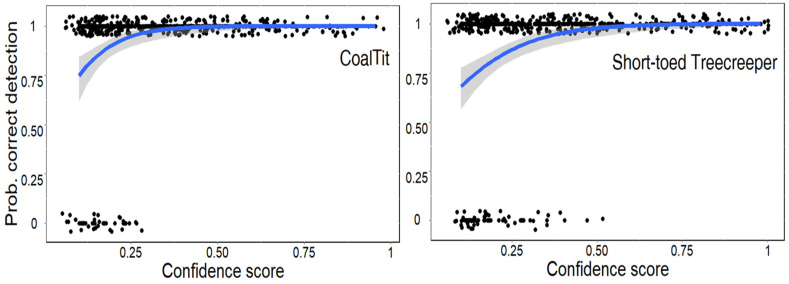
Results of the logistic regression showing the relationship between the probability of a correct BirdNET prediction and the confidence score of a given prediction for the (**left**) Coal Tit and the (**right**) Short-toed Treecreeper. Statistical analyses were performed using the BirdNET logit-scale of the prediction score (see Section 2) as an independent variable, but we represent the original confidence score of BirdNET for graphical purposes.

BirdNET precision was high for both the Coal Tit and the Short-toed Treecreeper, with mean values of 92.6% and 87.8%, respectively (Table 1). As expected, the confidence score threshold had a significant impact on the accuracy of bird vocalization to be correctly identified (Table 1, Figure 1). For the Coal Tit, the highest confidence score of a mislabeled detection was 0.267, indicating that all detections with a confidence score higher than this value were correctly classified. Similarly, for the Short-toed Treecreeper, the mislabeled detection with the highest confidence score had a value of 0.471. A summary table showing the overall BirdNET precision for both species across three confidence score categories can be seen in Table 1.

According to the logistic regressions the equations considering a 95% probability of correct identification for each species were:

logit(P Coal Tit) = ln(0.154) + 0.95 × ln(9.300), *p* < 0.001,

logit(P Short-toed Treecreeper) = ln(0.290) + 0.95 × ln(5.237), *p* < 0.001.

Therefore, the minimum confidence score to consider only detections with a 95% probability of correct identification was 0.247 for the Coal Tit and 0.335 for the Short-toed Treecreeper (Figure 1).

According to the validation dataset (100 15-min recordings), a human detected the presence of the Coal Tit and the Short-toed Treecreeper in 42 and 64 recordings, respectively (Table 2). When using BirdNET with the default confidence score the Coal Tit was detected in 38 recordings (90.5% of the recordings with known presence) while it was detected in 23 recordings (54.8%) using the optimal confidence score (Table 2). For the Short-toed Treecreeper, BirdNET correctly detected the species in 63 recordings (98.4% of the recordings with known presence) using the default confidence score and in 52 recordings (81.3%) using the optimal confidence score (Table 2). In both cases, there was a higher agreement between BirdNET and the human observer in terms of correctly predicting the presence or absence of each species when using the default confidence scores (95 and 92 recordings for the Coal Tit and Short-toed Treecreeper, respectively) compared to the optimal confidence score (81 and 86 recordings for the Coal Tit and Short-toed Treecreeper, respectively; see Table 2). However, the number of mislabeled recordings (i.e., where BirdNET annotated the presence of the species, but it was not confirmed) decreased from 1 to 0 when using the optimal confidence score instead of the default values for the Coal Tit and from 7 to 2 in the case of the Short-toed Treecreeper (Table 2).

### 3.2. Diel Vocal Activity Pattern

**Figure 2:**
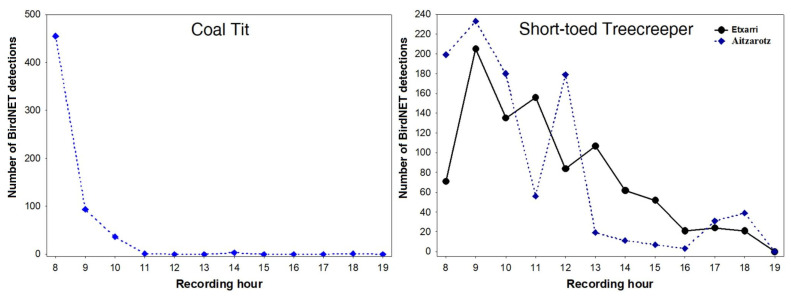
Diel pattern of vocal activity of the (**left**) Coal Tit and the (**right**) Short-toed Treecreeper in Northern Spain. Vocal activity was monitored using passive acoustic monitoring between 24 February and 23 March 2022 in two forested areas from 8:00 to 19:15. The first 15 min of each hour were recorded. The diel pattern is expressed as the total number of BirdNET detections with a 95% probability of correct identification per recording hour. The data shown is based on 568 BirdNET detections of the Coal Tit in Aitzarotz (data from Etxarri not shown in the graph since there were only 21 detections) and 1905 BirdNET detections (948 in Etxarri and 957 in Aitzarotz) of the Short-toed Treecreeper.

The vocal activity of both species is described using those detections with a 95% probability of correct identification (thresholds of confidence score of 0.247 and 0.335 for the Coal Tit and the Short-toed Treecreeper, respectively). The diel pattern of vocal activity differed between species. The vocal activity pattern of the Coal Tit occurred primarily during the first two hours of the day, with 96.1% of all BirdNET detections (566 out of 589) occurring at 8 a.m. and 9 a.m. (Figure 2). The peak vocal activity of the Short-toed Treecreeper, in both sites, also occurred at 9 a.m., accounting for 23.0% of the total predictions (Figure 2). However, unlike the Coal Tit, the Short-toed Treecreeper sustained a high level of vocal activity throughout the morning, gradually decreasing until reaching minimal levels by the end of the day (Figure 2).

## 4. Discussion

In this article, we have demonstrated the effectiveness of using low-cost open-source acoustic sensors in combination with BirdNET, a readily available machine learning tool, for efficient monitoring of two cryptic bird species in forest environments: the Coal Tit and the Short-toed Treecreeper. While the use of acoustic sensors mounted in ARUs is well-established for monitoring wildlife and ecosystems (see, e.g., [1,2,34]), the analyses of acoustic data has posed challenges in terms of automation and scalability. However, recent advancements have aimed to address these challenges, with BirdNET being a notable contribution in this field [11,19]. BirdNET is a convolutional neural-network-based tool designed for processing acoustic data [11]. Although BirdNET has rarely been used in scientific studies, the existing evaluations have consistently reported a high accuracy in identifying bird species (reviewed by [19]) but also anurans and primates [21,22].

We have demonstrated that BirdNET achieved a high level of precision in correctly identifying the Coal Tit and the Short-toed Treecreeper, with a mean precision of 93% and 88%, respectively, when using the default confidence score threshold. We also observed that the precision of BirdNET was highly varied with the selection of the confidence score threshold, with no mislabeled identifications when using a confidence score threshold of 0.247 for the Coal Tit and 0.335 for the Short-toed Treecreeper. Our findings are in agreement with [35], who also reported improved precision in BirdNET when using a higher confidence score threshold for three North American bird species. Although the impact of the confidence score on BirdNET output may vary among species, the general pattern is consistent, with larger precision values obtained when using a high confidence score, but it lowers the proportion of predictions made and, therefore, the proportion of calls and presences detected [19]. However, our current knowledge of the specific impact of different confidence scores on BirdNET’s ability to accurately detect species’ presence in sound recordings is very limited (but, see [19,35] and next paragraph).

In our study, we determined the optimal confidence score for each monitored species, which was defined as the minimum confidence score required to consider only detections with a 95% probability of correct identification. The defined values might be used for future studies aiming to monitor the Coal Tit or the Short-toed Treecreeper. We used these optimal values as thresholds to assess the impact of confidence score BirdNET’s ability to detect the presence of both species in sound recordings and to describe their diel pattern of vocal activity in a large acoustic dataset. As expected, when using the optimal confidence score, the number of detected presences decreased compared to using the default values. The percentage of presences detected by BirdNET using the optimal confidence score, instead of the default values, decreased more for the Coal Tit (from 90.5% to 54.8%) than for the Short-toed Treecreeper (from 98.4% to 81.3%). It is a surprising result since the optimal confidence score of the Short-toed Treecreeper was higher (0.335) than the one of the Coal Tit (0.247); therefore, a higher decrease in presences detected for the Short-toed Treecreeper would have been expected. One possible explanation for this surprising result might be related to a high vocal activity of the Short-toed Treecreeper, which may compensate for the potential decrease in the number of vocalizations detected when using a higher confidence score. Our findings might be also affected by different foraging strategies or territorial behavior of the target species and, therefore, by birds moving more often outside the detection range of the recorder. However, we were unable to include this factor in our analyses. In our study we focused on the ability of BirdNET to detect the species’ presence in sound recordings, but further research could expand on this by assessing the ability of BirdNET to detect vocalizations, an acoustic metric known as recall rate, which is not frequently evaluated in BirdNET surveys [19].

The selection of an appropriate confidence score in BirdNET may depend on the priorities of the user (e.g., ability and time to verify more or less false positives) and research goals. A recent review on BirdNET suggested starting with a minimum confidence score of around 0.5 to assess BirdNET performance [19], while other authors have recommended that confidence scores of 0.7–0.8 should be in the appropriate range for most studies [36]. However, our study highlights the importance of conducting species-specific assessments (e.g., by creating independent validation datasets for each species) for choosing an appropriate confidence score and how this selection can vary depending on research goals. According to our data, if our objective is to detect the presence of both species in sound recordings it would be necessary to use low confidence scores (e.g., 0.1, default values). Otherwise, a significant number of presences will be undetected. On the other hand, if our goal is to study ecological processes, which usually require low-error estimates, such as describing the vocal behavior of a bird species, selecting a higher confidence value may be more appropriate. Although this selection may decrease the number of vocalizations detected, it would provide a more reliable description of the behavior (see [21]).

Finally, we linked BirdNET detections to ecological processes using a large acoustic dataset as an example of how this tool may help researchers and managers to improve acoustic monitoring programs and contribute to a better understanding of the ecological processes. The vocal activity of the Coal Tit was primarily concentrated in the first two hours after sunrise, with minimal vocal activity throughout the day. This pattern aligns with the typical vocal activity pattern observed in most passerines [37,38], and with the pattern described for two close-related species, the Blue Tit (*Cyanistes caeruleus*) and the Great Tit (*Parus major*) [39]. The vocal activity of the Short-toed Treecreeper, similar to the Coal Tit, peaked during the first hours after sunrise. However, the Short-toed Treecreeper showed sustained vocal activity throughout the morning, and the species even vocalized during the afternoon. This prolonged vocalization behavior might be related to the species’ strong vocal response towards other species, particularly to the closely related Common Treecreeper (*Certhia familiaris*, [40,41]), which coexists in the study area and may have stimulated the vocal activity of the Short-toed Treecreeper.

## 5. Conclusions

Our study has demonstrated the effectiveness of BirdNET, a new tool for processing acoustic data, in accurately identifying two cryptic bird species and detecting their presence in sound recordings. We have also highlighted the importance of carefully selecting the confidence score, as it has a significant impact on the output of BirdNET, and may potentially lead to poorly informed conclusions. In both species a higher confidence score reduces false positives but also results in fewer detections of species’ presences. We hope that our assessment and the methods we employed, including calculating the optimal confidence score (see [22]), will encourage researchers and managers to make use of this freely available software (accessible on GitHub) that is user-friendly (e.g., can be run as a GUI from Windows) and ready-to-use (>6500 species already included). Additionally, the continuous development of BirdNET (last update in June 2023) will contribute to further improvements in acoustic sensing and monitoring in both urban and natural environments, including the ability to detect multiple species from various taxa simultaneously [19,20,21,22].

## Figures and Tables

**Table 1:** Number of BirdNET detections (annotated by the software) and verified detections (correctly classified after human verification) for the Coal Tit and the Short-toed Treecreeper across three confidence score categories. The overall precision of BirdNET (in percentage, %) for each category and species is shown between brackets.

Confidence Score Category	Coal Tit		Short-Toed Treecreeper	
	Detections	Verified	Detections	Verified
(0.1–0.29)	157	134 (85.3%)	153	119 (77.8%)
(0.3–0.49)	77	77 (100%)	77	73 (94.8%)
>0.5	75	75 (100%)	81	81 (100%)
TOTAL	309	286 (92.6%)	311	273 (87.8%)

**Table 2:** Confusion matrix of the ability of BirdNET to correctly detect the presence of the Coal Tit and the Short-toed Treecreeper in sound recordings. The validation dataset was composed of 100 15-min recordings manually reviewed for each species, in which the Coal Tit and the Short-toed Treecreeper were known to be present in 42 and 64 recordings, respectively.

	Coal Tit				Short-Toed Treecreeper			
	Default Values (>0.1)		Optimal Score (>0.247)		Default Values (>0.1)		Optimal Score (>0.335)	
	Detected	Not-Detected	Detected	Not-Detected	Detected	Not-Detected	Detected	Not-Detected
Presence	38	4	23	19	63	1	52	12
Absence	1	57	0	58	7	29	2	34

## Data Availability

Raw databases employed for BirdNET validation can be downloaded at: https://figshare.com/s/888467a40c77f46d463a, with https://doi.org/10.6084/m9.figshare.23736570, accessed on 11 August 2023.
